# Altered post-fracture systemic bone loss in a mouse model of osteocyte dysfunction

**DOI:** 10.1093/jbmrpl/ziae135

**Published:** 2024-11-01

**Authors:** Benjamin Osipov, Armaun J Emami, Hailey C Cunningham, Sophie Orr, Yu-Yang Lin, Elias H Jbeily, Ritvik S Punati, Deepa K Murugesh, Hannah M Zukowski, Gabriela G Loots, Randy Carney, Diego Vargas, Virginia L Ferguson, Blaine A Christiansen

**Affiliations:** Department of Orthopaedic Surgery, University of California Davis Medical Center, Sacramento, CA 95817, United States; Department of Orthopaedic Surgery, University of California Davis Medical Center, Sacramento, CA 95817, United States; Department of Orthopaedic Surgery, University of California Davis Medical Center, Sacramento, CA 95817, United States; Department of Orthopaedic Surgery, University of California Davis Medical Center, Sacramento, CA 95817, United States; Department of Orthopaedic Surgery, University of California Davis Medical Center, Sacramento, CA 95817, United States; Department of Orthopaedic Surgery, University of California Davis Medical Center, Sacramento, CA 95817, United States; Department of Orthopaedic Surgery, University of California Davis Medical Center, Sacramento, CA 95817, United States; Physical and Life Sciences Directorate, Lawrence Livermore National Laboratories, Livermore, CA 94550, United States; Department of Orthopaedic Surgery, University of California Davis Medical Center, Sacramento, CA 95817, United States; Department of Orthopaedic Surgery, University of California Davis Medical Center, Sacramento, CA 95817, United States; Physical and Life Sciences Directorate, Lawrence Livermore National Laboratories, Livermore, CA 94550, United States; Department of Biomedical Engineering, University of California Davis, Davis, CA 95616, United States; Department of Mechanical Engineering, University of Colorado Boulder, Boulder, CO 80309, United States; Department of Mechanical Engineering, University of Colorado Boulder, Boulder, CO 80309, United States; Department of Orthopaedic Surgery, University of California Davis Medical Center, Sacramento, CA 95817, United States

**Keywords:** fracture, osteocytes, osteoporosis, bone remodeling, mouse models

## Abstract

Femur fracture leads to loss of bone at uninjured skeletal sites, which may increase risk of subsequent fracture. Osteocytes, the most abundant bone cells, can directly resorb bone matrix and regulate osteoclast and osteoblast activity, but their role in systemic bone loss after fracture remains poorly understood. In this study we used a transgenic (TG+) mouse model that overexpresses human B-cell lymphoma 2 (BCL-2) in osteoblasts and osteocytes. This causes enhanced osteoblast proliferation, followed by disruption in lacunar-canalicular connectivity and massive osteocyte death by 10 wk of age. We hypothesized that reduced viable osteocyte density would decrease the magnitude of systemic bone loss after femur fracture, reduce perilacunar remodeling, and alter callus formation. Bone remodeling was assessed using serum biomarkers of bone formation and resorption at 5 d post-fracture. We used micro-computed tomography, high resolution x-ray microscopy, mechanical testing, and Raman spectroscopy to quantify the magnitude of systemic bone loss, as well as changes in osteocyte lacunar volume, bone strength, and bone composition 2 wk post-fracture. Fracture was associated with a reduction in circulating markers of bone resorption in non-transgenic (TG-) animals. TG+ mice exhibited high bone mass in the limbs, greater cortical elastic modulus and reduced post-yield displacement. After fracture, TG+ mice lost less trabecular bone than TG- mice, but conversely TG+ mice exhibited trends toward a lower yield point and reduced femoral cortical thickness after fracture, though these were not statistically significant. Lacunar density was greater in TG+ mice, but fracture did not alter lacunar volume in TG+ or TG- mice. These findings suggest that osteocytes potentially play a significant role in the post-traumatic systemic response to fracture, though the effects differ between trabecular and cortical bone.

## Introduction

Osteoporotic fracture significantly increases mortality^1^^,^^2^ and an estimated 2 million osteoporotic fractures occur annually in the United States.^3^ While fracture has a complex etiology, a first (index) fracture increases future fracture risk 2-10-fold at all post-cranial skeletal sites.^4^^,^^5^ While some portion of increased risk likely stems from detrimental effects of the injury on physical function,^6^ a clinical study in our lab found that incident upper or lower body fracture in women aged >65 yr was associated with accelerated decline of hip BMD during the 2 yr that included the fracture.^7^ We have also shown that femur fracture induces systemic bone loss during the first 2 wk post-fracture in young (12-wk-old) and middle aged (52-wk-old) female mice.^8^ Post-traumatic bone loss was associated with systemic inflammation at 1 d post-fracture and reduced activity during the first week post-fracture.^9^ By 6 wk post-injury, young mice recovered to baseline values, whereas bone mineral density did not recover in middle-aged animals.^8^ A subsequent study showed that bone loss was significantly greater in male than female 3-mo-old mice.^9^

Osteocytes play a key role in mechanotransduction and bone remodeling, but their role in post-fracture systemic bone loss remains poorly understood.^10^ Derived from osteoblasts embedded in extracellular matrix, osteocytes are the most abundant bone cells. Via lacunae and canaliculi, osteocytes contact over 100 times more bone surface than osteoclasts or osteoblasts.^11^^,^^12^ Osteocytes can directly resorb surrounding bone matrix through a process known as perilacunar remodeling (PLR) in which osteocytes secrete metalloproteinases (MMPs), Cathepsin K, carbonic anhydrase 2, and tartrate resistant acid phosphatase.^13^^,^^14^ PLR was originally identified as a response to physiological stressors (eg, lactation, deficiency of vitamin D, phosphate and calcium deficiency, unloading or ovariectomy).^15^^,^^16^ However, a growing number of studies have demonstrated that PLR also plays an essential role in regular bone homeostasis and maintaining bone material properties, such as degree of inorganic matrix mineralization and crystallinity.^13^^,^^17^ Osteocytes also secrete regulators of osteoblast and osteoclast mediated remodeling including receptor activator of NF-κB ligand (RANKL), osteoprotegerin (OPG), and sclerostin. In fracture healing, osteocytes may coordinate the initial inflammatory response, as well as creation and remodeling of the callus^18^ Our prior study of the role of osteocytes in systemic bone loss found significant increases in canalicular width in contralateral tibia 2 and 4 wk post-fracture.^19^ Also, 3 d post fracture, bulk RNAseq of cortical bone showed a downregulation of genes associated with bone metabolism. Thus prior work has established that osteocytes are master regulators of bone remodeling, and they may directly contribute to systemic bone loss after fracture.

In this study, we further explore the role of osteocytes in systemic bone loss after fracture, employing the BCL-2 transgenic mouse model established by Moriishi et al.^20^^,^^21^ These mice overexpress human B-cell lymphoma 2 (BCL-2) in osteoblasts and osteocytes, under the control of the Col1a1(2.3 kb) promoter. BCL-2 transgenic mice exhibit increased osteoblast proliferation and enhanced osteoblast function, as evidenced by increased trabecular and cortical bone mass in the appendicular but not the axial skeleton by 4 mo of age. Osteocytes exhibit fewer and shorter cell processes, resulting in a disruption of the canalicular network. Death of 50% of all osteocytes occurs by 10 wk of age and 75% by 16 wk of age.^22^

In this study, we investigated the effect of osteocyte deficiency on systemic bone loss after fracture using BCL-2 transgenic mice (TG+) and Wild Type (TG-) littermate controls. Because we have previously shown evidence of systemic PLR post-fracture, we hypothesized that systemic bone loss would be diminished, and that there would be less change in markers of bone turnover after fracture in TG+ mice compared to TG- animals. We further hypothesis that osteocyte lacunar volume would increase in the contralateral (non-fractured) limb of TG- fracture animals compared to TG- controls, but lacunar volume would not vary due to fracture in TG+ animals. We also hypothesized that both transgene and fracture would cause changes in bone chemical composition, due to altered PLR as well as altered osteoblast and osteoclast mediated bone turnover. Lastly, we hypothesized that transgene expression would be associated with diminished callus formation during bone healing.

## Materials and methods

### Animals and study design

This study used a total of 70 mice. For fractures we used 66 3-mo-old female mice (35 BCL-2 TG+ animals and 29 female TG- littermate controls bred at Lawrence Livermore National Laboratory, Livermore, CA). We also harvested tibia from 4 12-wk-old male (2 TG+ and 2 TG-) for preliminary bone phenotyping of this strain. Breeding pairs of BCL-2 TG+ mice were provided to us by Dr Toshihisa Komori, Nagasaki University, Nagasaki, Japan and generated from a C57BL/6 x C3H-HEJ hybrid. Female mice were used to make the study directly comparable to our initial work documenting systemic bone loss in female mice.^8^ Since our previous studies were on C57BL/6 mice, we assess if the TG- littermates showed different systemic responses to fracture than observed in our previous studies in the results and discussion below. We used 3-mo-old animals, because older TG- female mice have insufficient trabecular bone for analysis of the distal femoral metaphysis. Mice were cared for in accordance with the guidelines set by the National Institutes of Health (NIH) on the care and use of laboratory animals. All procedures were approved by the Institutional Animal Care and Use Committee at UC Davis.

Animals were allowed to acclimate to the vivarium for 2 wk prior to surgery. Mice had ad libitum access to food (Harlan irradiated 2918 chow) and autoclaved water and were monitored by husbandry staff at least once a day. TG+ and TG- animals were randomly assigned to fracture and control groups. Groups of mice (7 TG- Cntrl, 8 TG- FX, 7 TG+ Cntrl, 7 TG+ FX) were euthanized at 14 d post-fracture for assessment of trabecular and cortical bone. This time point was chosen since it was the time at which peak post-fracture bone loss was observed in our previous studies. A further cohort of mice was used for blood collection 5 d after fracture (7 TG- Cntrl, 9 TG- FX, 9 TG+ Cntrl, 12 TG+ FX). Initially, they were slated for sacrifice at 6 wk post-fracture, but this timepoint could not be met due to COVID-related lab shutdown in 2020. A total of 5 mice were euthanized for poor pin placement or other surgical complications during femur fracture creation (described below).

### Creation of femur fracture

All mice used for fracture surgery received a dose of buprenorphine (0.05 mg/kg subcutaneous injection) pre-operation and mice were anesthetized via inhalation of 2-4% isoflurane in oxygen. Fur was removed from the anterior leg and thigh with a depilatory cream. Femur fractures were induced as originally described by Bonnarens and Einhorn^23^ on the right leg. An incision was created on the medial side of the knee and a 0.25 mm stainless steel wire pin was inserted into the medullary canal. Transverse fractures were then created with a controlled lateral impact using an electromagnet drop-weight fracture apparatus. Control mice received anesthesia and analgesia only, with no surgical procedures. Immediately post-fracture, mice were imaged with planar radiographs (HE100/30þX-ray machine, MinXRay, Northbrook, IL, United States) with a CXD1-31 plate (Canon, LakeSuccess, NY, United States) to confirm pin positioning and a transverse mid-diaphyseal fracture. Five mice were euthanized immediately after fracture due to pin misplacement. Full weight-bearing and unrestricted activity was permitted postoperatively. All mice received an additional dose of buprenorphine (0.05 mg/kg) 8 hours post-operation, and then twice daily for 2 d thereafter.

### Collection of serum and quantification of bone turnover markers

Non-terminal blood draw was conducted via retro-orbital bleeding at 5 d post-fracture. We selected this time point, because we previously observed enhanced osteoclast activity at uninjured skeletal sites in the first week after facture in female mice.^8^ Mice were anesthetized with isoflourane, and proptosis of the eye was induced. A sterile Pasteur pipette was then inserted at the medial canthus of the eye, and the retro-orbital sinus was punctured with gentle pressure and twisting of the pipette.^24^ Approximately 200-300 μL of blood was collected, and mice were given 500 μL of saline subcutaneously for recovery. Blood was allowed to clot for 1 hour and then centrifuged at 1500 gravities at 4°C for 10 min. Serum was transferred to a new Eppendorf tube and stored at −80°C prior to analysis. Commercially available ELISA kits (CUSABIO) were used to assay Procollagen type 1 N-terminal Propeptide (P1NP) as a marker for bone formation and C-terminal telopeptide of type I collagen (CTX-1) as a marker of bone resorption.

### Histology for bone phenotyping

Four 12-wk-old male mice were euthanized at 12 wk of age for preliminary bone phenotyping of the BCL2 strain. Following euthanasia, tibia were fixed in a 4% paraformaldehyde (PFA) solution for 3 d and then stored in 70% ethanol until decalcification. Tibias were decalcified with 0.5 M EDTA until fully decalcified as determined via x-ray. Decalcified tibias were processed for paraffin embedding and 6 μm sagittal sections were cut from the mid diaphysis to proximal end of the tibia. Slides were then deparaffinized, rehydrated, and stained with H&E.

### Micro-computed tomography of trabecular and cortical bone and fracture callus

After euthanasia, L5 vertebrae and fractured and contralateral femora were removed, fixed in 4% PFA, and preserved in 70% ethanol. Bones were then scanned using micro-computed tomography (SCANCO Medical, μCT 35, Brüttisellen, Switzerland) with 6 mm nominal voxel size (X-ray tube potential = 55 kVp, current = 114 mA, integration time = 900 ms, number of projections = 1000/180°) according to the JBMR guidelines for μCT analysis of rodent bone structure.^25^ All analyses were performed using the manufacturer’s analysis software. Trabecular bone was analyzed at the L5 vertebral body and distal femoral metaphysis. Trabecular ROIs were manually drawn on transverse images excluding the cortical surface. The ROI for the L5 vertebral bodies extended from the cranial growth plate to the caudal growth plate, excluding the transverse processes. The ROI for the distal femoral metaphysis started adjacent to the metaphyseal growth plate and extended 250 slices proximally (1500 μm). Trabecular bone volume fraction (BV/TV), trabecular thickness (Tb.Th), trabecular number (Tb.N), trabecular spacing (Tb.Sp), apparent BMD, and bone tissue mineral density (TMD) were determined using the manufacturer’s analysis software. Analysis of cortical bone in the femoral diaphysis was performed by contouring transverse slices, with a 1200 μm (200 slices) ROI centered on the midpoint of the femur. Bone area (B.Ar), cortical thickness (Ct.Th), bone TMD, and other microstructural parameters were determined using the manufacturer’s analysis software. Fracture callus total volume, bone volume, BV/TV, and other microstructural parameters were quantified using a 6000 um ROI centered on the midpoint of the fracture callus.

### 3-point bending of contralateral/ uninjured femora

3-point bending was performed on femora to determine structural and material properties using a material testing system (ELF 3200, TA Instruments, New Castle, DE, United States). Left femora were rehydrated in PBS solution prior to testing. The span length of the lower supports was 8 mm, and the femur was positioned so that the anterior aspect of the midshaft was loaded in tension. A 1-2 N preload was applied to ensure contact with the upper platen, then loading was applied at a displacement rate of 0.01 mm/sec until fracture. Resulting force and displacement data were recorded at 50 Hz and analyzed to determine whole bone stiffness, yield force, ultimate force, energy to fracture, and post-yield displacement. Material properties of the femur diaphysis were calculated using structural measurements of the femoral midshaft determined with μCT using previously established beam theory equations.^26^

### Raman spectroscopy analysis of cortical bone composition

Raman spectra were acquired on the periosteal surface of the femoral midshaft after 3-point bending mechanical testing. We used the largest half of the femur diaphysis remaining after mechanical testing and sampled periosteal bone at 10 randomly selected locations within approximately 2 mm of the break on each bone. Spectra were acquired using a custom-built inverted Raman scanning confocal microscope with excitation wavelength of 785 nm and a 60×, 1.2 NA water immersion objective on an inverted IX73 Olympus microscope. An Andor Kymera-3281-C spectrophotometer and Newton DU920P-BR-DD CCD camera were used for Raman spectra capture and Solis v4.31.30005.0 software was used for initial processing. For measurements, exposure time was 180 s per scan with laser power of 65 mW and an aperture of 2 mm. The spectral range was 300–1800 cm^−1^. Penalized least-squares background correction and smoothing of the obtained Raman spectral data, as well as cosmic ray removal was performed via custom MATLAB scripts (MathWorks, Natick, MA, United States). We identified Raman peaks for phosphate ν1 (960 cm^−1^), carbonate (1069 cm^−1^), CH_2_ (1448 cm^−1^), amide I (1664 cm^−1^), and amide III (1215 cm^−1^).^27^ Following prior studies, ratios of carbonate/phosphate, phosphate ν1/CH_2_, phosphate ν1/amide III were calculated from peak intensities.^28–30^ The ratio of carbonate to phosphate measures changes in the inorganic component of bone matrix. The mineral to organic matrix ratio was assessed using the phosphate ν1/amide III ratio. Other ratios commonly used for this assessment produced the same results. The amide I/CH_2_ ratio indicates the relative abundance of matrix protein to all organic matrix components. We also calculated maximum width at half maximum height of the primary phosphate band as a measure of mineral crystallinity.

### High resolution X-ray microscopy of lacunar morphology

X-ray microscopy (XRM) was used to evaluate the morphological characteristics of osteocyte lacunae in the proximal tibia (Xradia Versa XRM-520, Zeiss, Dublin, CA) following previously published methodologies.^31^ Tibiae from the contralateral (non-fractured) limb were harvested, and all non-osseous tissue was removed. Samples were mounted in a custom sample holder that oriented the samples vertically on the stage using superglue and allowed to air-dry for ~48-72 hours to ensure minimal scanning drift. Scans with a 4× objective were used to determine ROIs. For cortical bone, a 650 μm^3^ region of bone was located on the anterior medial aspect of the tibia, 5 mm from the tibiofibular junction. The trabecular bone ROI was placed 2 mm distal to the proximal epiphysis. Nano-computed tomography images were collected in the ROI using a 20× objective with energy settings of 50 kV, 4.0 W, using the Air Filter and 1601 projections. To obtain a constant resolution of 0.6 μm voxel for all the samples, the source and detector distance were varied between 5.8 and 8.5 mm from the sample, and excitation ranged from 6 to 8 s contingent on resulting intensity values.

Dragonfly 3.6 (Object Research Systems (ORS) Inc, Montreal, QC) was used for segmentation of osteocyte lacunae regions. Cortical and trabecular bone were manually segmented. Osteocyte lacunae were identified and segmented in 3 dimensions using a global Otsu threshold, defined for each sample to most closely match grayscale images. Voids that fell outside the size range of osteocyte lacunae (<50 μm^3^ and >1500 μm^3^) were removed. Osteocyte volume, surface area, and number were measured. Lacunar density was calculated as the number of lacunae per μm^3^.

### Statistical methodology

All data were tested for normality of distribution using a Shapiro–Wilk test. As all data were normally distributed, a 2-way ANOVA was employed with genotype and fracture as factors and Tukey Honest Significant Differences test used for post hoc comparisons.

## Results

### Bone phenotyping

As a preliminary examination of bone organization, we performed H&E staining of tibial cortical bone in 12-wk-old male TG+ and TG- littermates collected for a prior study. Representative images are presented in Figure S1. There was no difference in lamellar bone organization. As expected, osteocyte lacuna showed open space indicative of osteocyte apoptosis in TG+ mice. Further, consistent with our prior study, we also observed a greater prevalence of large vascular canals in TG+ mice.^32^

### Bone microstructural properties

**Figure 1:**
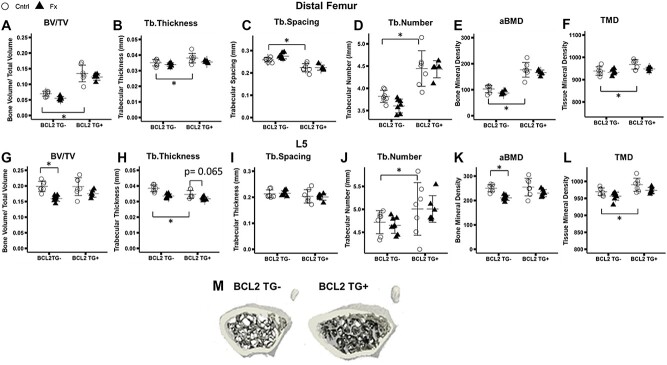
Micro-computed tomography analysis of fifth lumbar vertebrae (L5) and contralateral (uninjured) distal femur trabecular bone. Group sizes: L5: TG- Cntrl: 7, TG- Fx: 8, TG+ Cntrl: 7, TG+ Fx: 6; distal femur: TG- Cntrl: 7, TG- Fx: 7, TG+ Cntrl: 7, TG+ Fx: 5. (A-F) Distal femur microarchitectural properties. Significant differences are evident between TG+ and TG- animals. (G-L) Lumbar spine trabecular microarchitectural properties. Significant decreases are seen in TG- fracture relative to control mice, but the same effect of fracture is not evident in TG+ mice (G-L). (m) Representative images of BCL2 TG- and TG+ distal femur metaphysis. ^*^p < 0.05. Abbreviations: BCL2, B-cell lymphoma 2; TG-, non-transgenic; TG+, transgenic.

At the distal femur, BCL-2 TG+ mice exhibited 93% greater trabecular bone volume fraction (BV/TV) (Figure 1A), 8% greater trabecular thickness (Tb.Th) (Figure 1B), 14% reduction in trabecular separation (Tb.Sp) (Figure 1C), 16% greater trabecular number (Tb.N) (Figure 1D), 72% greater apparent bone mineral density (aBMD) (Figure 1E), and 3% greater bone TMD (Figure 1F) compared to TG- mice (BV/TV*Genotype: *p* < 0.001, Tb.Sp*Genotype: *p* < 0.001; Tb.N*Genotype: *p* < 0.001; aBMD*Genotype: *p* < 0.001; TMD*Genotype: *p* < 0.001). The same difference in BV/TV, Tb.Sp, and aBMD was not observed between TG+ and TG- animals at the L5 vertebra (Figure 1G,I, K). At this skeletal site, TG- animals exhibited 10% greater Tb.Th (*p* = 0.001) (Figure 1H) and 6% greater Tb.N (*p* = 0.022) (Figure 1J), but TMD was 2% higher in TG+ animals (*p* < 0.001) (Figure 1L). Representative images of the distal femur trabecular in TG+ and TG- mice are given in Figure 1M, demonstrating the greater trabecular and cortical bone mass of TG+ mice in the femur.

Fracture was associated with declines in BV/TV and Tb.Th relative to genotype-matched controls in both the lumbar spine and distal femur (L5: Fracture: BV/TV: *p* < 0.001; Tb.Th*FX: *p* < 0.001; aBMD*FX: *p* < 0.001; TMD*FX: *p* = 0.008; Distal Femur: Fracture: BV/TV*FX: *p* = 0.046; Tb.Th*FX: *p* = 0.039; aBMD*FX: *p* = 0.061; TMD*FX: *p* = 0.068) (Figure 1). The 2-way ANOVA interaction between genotype and fracture was not significant, but post hoc comparisons detected significant differences between fracture and control mice in TG- but not TG+ mice. TG+ mice lost approximately half as much trabecular bone as TG- mice at the lumbar spine. BV/TV was 19% lower (*p* = 0.002) in TG- fractured mice compared to TG- controls but decreased 11% in the TG+ fracture group versus genotype-matched controls (*p* = 0.131) (Figure 1G). Similarly, Tb.Th decreased by 13% (*p* < 0.001) and aBMD by 16% (*p* = 0.008) in TG- fracture animals, whereas Tb.Th decreased by 8% (*p* = 0.065) and aBMD decreased by 10% (*p* = 0.181) in fractured TG+ animals (Figure 1H,K). Post hoc comparisons at the distal femur did not find significant differences between fracture and control in either TG- or TG+ groups, but TG- fracture animals still exhibited a 21% lower BV/TV than unfractured TG- animals, compared to an 8% decrease in TG+ animals relative to TG+ controls (Figure 1A).

**Figure 2:**
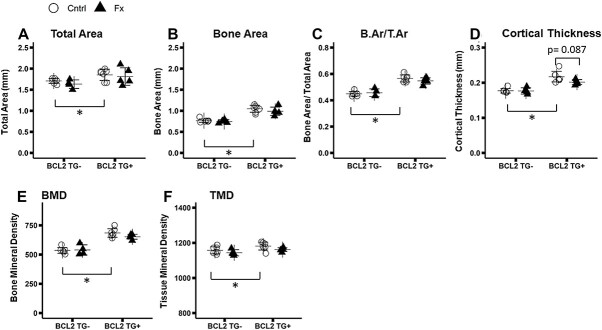
Micro-computed tomography analysis of contralateral (uninjured) femur midshaft cortical bone. Group sizes: TG- Cntrl: 7, TG- Fx: 4, TG+ Cntrl: 7, TG+ Fx: 5. (A-F) Femur cortical microarchitectural properties. BCL2 TG+ mice exhibit higher bone area and increased density. BCL2 TG+ mice exhibit a trend toward decreased cortical thickness with fracture, which is not evident in TG- mice (B) ^*^p < 0.05. Abbreviations: BCL2, B-cell lymphoma 2; TG-, non-transgenic; TG+, transgenic.

**Figure 3:**
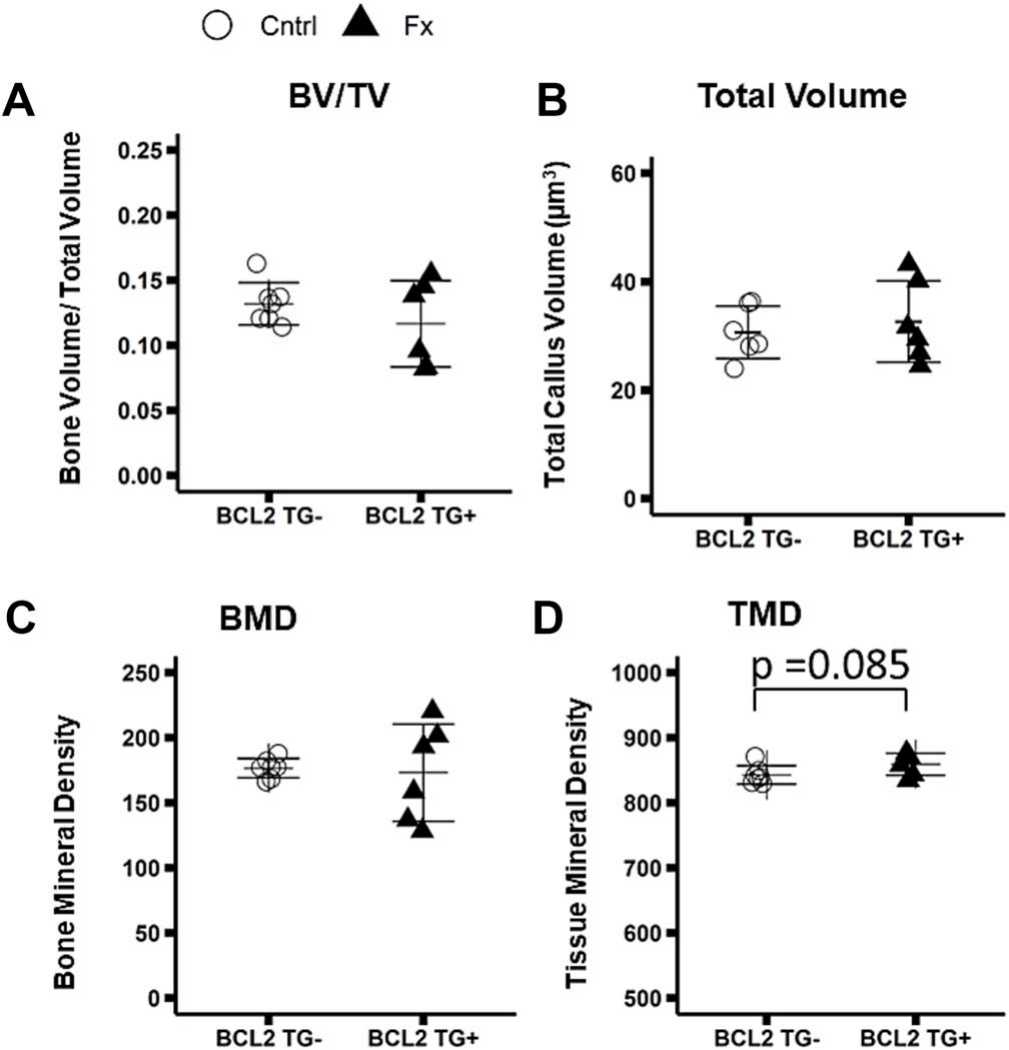
Micro-computed tomography analysis of fracture callus at the femur diaphysis. Group sizes: TG- Fx: 7, TG+ Fx: 7. (A-D) Microarchitectural properties of the fracture callus. There is no difference evident between TG- and TG+ mice, except a trend toward greater tissue mineral density in BCL2 TG+ animals. ^*^p < 0.05. Abbreviations: BCL2, B-cell lymphoma 2; TG-, non-transgenic; TG+, transgenic.

At the femoral midshaft, total bone area, cortical bone area, bone area fraction (B.Ar/T.Ar), cortical thickness, and TMD were between 23 and 28% greater in TG+ than non-transgenic animals (Total Area*Genotype: *p* < 0.001; Cortical Area*Genotype: *p* < 0.001; Ct.Th*Genotype: *p* < 0.001; TMD*Genotype: *p* = 0.017) (Figure 2A-F). TMD decreased significantly in fracture mice (TMD*FX: *p* = 0.047), and there was a trend toward decreased Ct.Th in fractured animals (Ct.Th*FX: *p* = 0.072) (Figure 2D,F). While post hoc comparisons did not reach statistical significance, there was a trend toward greater differences between fracture and control animals in the TG+ group. Ct.Th was 7% lower in TG+ Fracture than TG+ Control animals (*p* = 0.087), whereas the difference was only 0.3% in TG- animals (*p* = 0.999) (Figure 2D). Callus size, bone content, and mineralization did not differ between genotypes (Figure 3).

### 3-point bending mechanical testing of femoral midshaft

**Figure 4:**
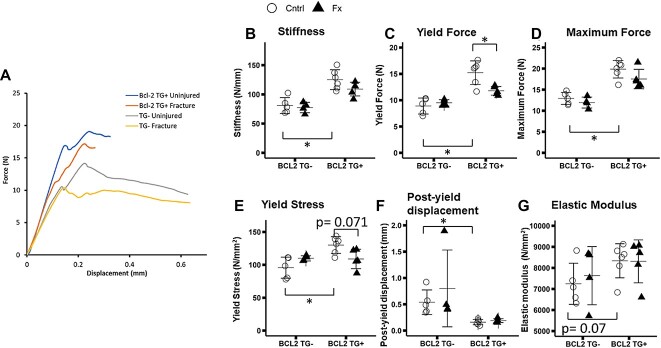
Whole bone and tissue level mechanical properties derived from 3 point bending tests of the contralateral (uninjured) femur. Group sizes: TG- Cntrl: 5, TG- Fx: 4, TG+ Cntrl: 6, TG+ Fx: 5. (A) Representative force displacement curves grouped by genotype and fracture status. Whole bone mechanical and tissue level properties (B-G) Tissue level mechanical properties. BCL2TG+ animals exhibit stiffer bones with lower post yield displacement. BCL2 TG+ fracture mice exhibit decreased yield force and stress with fracture, unlike TG- mice (C,E). ^*^p < 0.05. Abbreviations: BCL2, B-cell lymphoma 2; TG-, non-transgenic; TG+, transgenic.

Representative force displacement curves are shown in Figure 4A. Femora from unfractured TG+ mice exhibited a 54% greater stiffness (*p* < 0.001), 70% greater yield force (*p* < 0.001), 54% greater maximum force (*p* < 0.001), and a 70% lower post yield displacement (*p* < 0.001) relative to femora from TG- mice (Figure 4B-G). Statistically significant 2-way interactions were observed between fracture and genotype for yield force and yield stress (Yield force TG*FX: *p* = 0.010; Yield Stress TG*FX: *p* = 0.009). TG+ fractured mice showed a 23% decrease in yield force (*p* = 0.009) and a non-significant trend toward a 16% decrease in yield stress (*p* = 0.071) (Figure 4C, E) relative to TG+ controls, whereas TG- fracture mice did not show decreases in these properties compared to TG- controls.

### Biomarkers of bone formation and resorption

**Figure 5:**
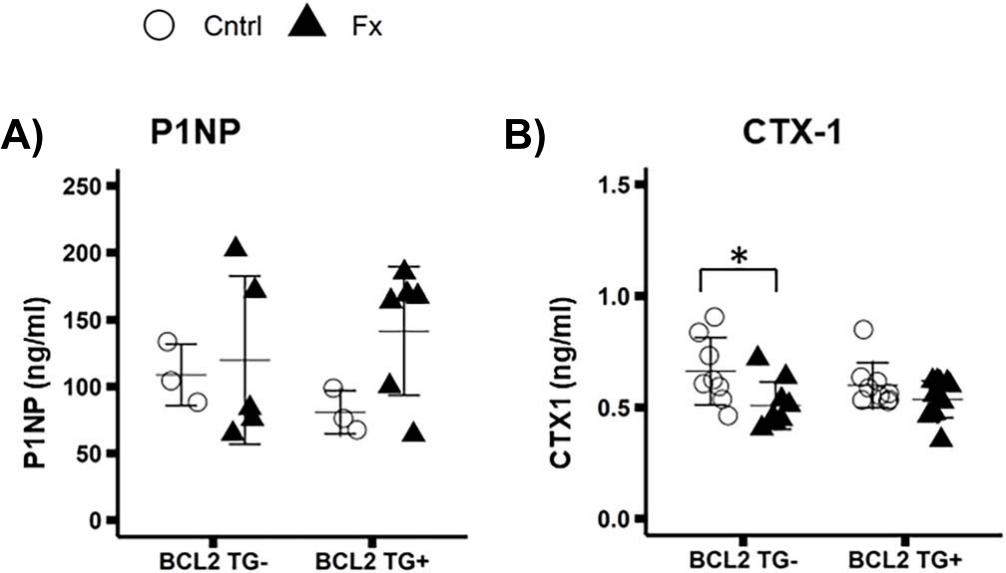
Serum biomarkers of bone remodeling collected 5 d post-fracture. Group sizes: P1NP: TG- Cntrl: 3, TG- Fx: 5, TG+ Cntrl: 3, TG+ Fx: 6; CTX-1: TG- Cntrl: 8, TG- Fx: 7, TG+ Cntrl: 9, TG+ Fx: 8. (A) Procollagen 1 N-terminal peptide (P1NP). P1NP does not differ due to genotype or fracture. (B) C terminal peptide (CTX-1) CTX-1 does not differ significantly due to genotype, but shows reductions in BCL2 TG- control mice relative to fracture mice, while there is no difference between TG+ control and fracture mice. ^*^p < 0.05. Abbreviations: BCL2, B-cell lymphoma 2; P1NP, Procollagen type 1 N-terminal Propeptide; TG-, non-transgenic; TG+, transgenic.

At 5 d post-fracture, serum levels of P1NP, the marker of bone formation, and CTX-1, the marker of bone resorption, did not differ significantly between TG+ and TG- mice (P1NP*TG: *p* = 0.902; CTX1*TG: *p* = 0.638) (Figure 5A,B). P1NP also did not show significant differences due to fracture, but CTX-1 was significantly lower in fracture mice (P1NP*FX: *p* = 0.159; CTX1*FX: *p* = 0.005). While the 2-way interaction of fracture and genotype were not significant, CTX-1 was 23% lower in TG- FX than TG- Cntrl mice compared to only a 10 % reduction due to fracture in TG+ animals. Post hoc comparisons of CTX-1 indicate a significant difference between TG- Cntrl and FX mice, but not TG+ Cntrl and FX mice (TG- Cntrl v FX *p* = 0.031; TG+ Cntrl v FX *p* = 0.559).

### X-ray microscopy of lacunar volume

**Figure 6:**
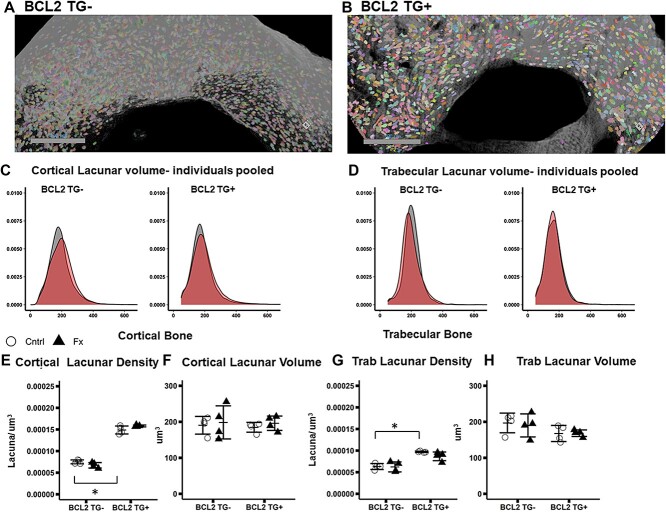
X-ray microscopy imaging of lacunar volume and density in contralateral (uninjured) tibia. Group sizes: TG- Cntrl: 4, TG- Fx: 4, TG+ Cntrl: 4, TG+ Fx: 4. (a-b) Representative images of tibial cortical bone ROI for (A) BCL2 TG- and (B) BCL2TG+. (C) Cortical lacunar volume by genotype and fracture. (D) Trabecular lacunar volume by genotype and fracture. (E-H) Lacunar density and average lacunar volume for cortical and trabecular bone ROIs. BCL2 TG+ mice have higher lacunar density but average lacunar volume does not differ by genotype (E-H). ^*^p < 0.05. Abbreviations: BCL2, B-cell lymphoma 2; TG-, non-transgenic; TG+, transgenic.

Representative images of cortical bone with segmented lacunae are shown in Figure 6A,B. Lacunar density was significantly greater (*p* < 0.001) in TG+ than TG- mice in both cortical and trabecular bone, however lacunar volume did not significantly different (Figure 6E-H). While there was no significant effect due to fracture, a significant interaction was detected between genotype and fracture for cortical bone (*p* = 0.022) but not trabecular bone lacunar density. The cortical bone interaction is driven by slightly increase lacunar density in TG+ fracture animals and decreases in TG- fracture animals. However, post hoc comparisons of fracture and control animals are not significant (Figure 6E). When all osteocytes are pooled across study groups, there is a trend toward a higher mean cortical lacunar volume and lower trabecular lacunar volume in fracture TG- mice compared to control (Figure 6C,D). However when average values for each mouse are used, lacunar volume did not differ significantly due to genotype or fracture in cortical bone (Figure 6F,H).

### Raman spectroscopy of bone chemical composition

Raman spectroscopy of the femoral midshaft did not detect differences in matrix composition due to genotype or fracture. There were no significant differences in inorganic matrix composition (Pv1/CO3), the ratio of inorganic to organic matrix components (Pv1/ Amide III), ratio of protein to all organic (Amide 1/ CH_2_) or mineral crystallinity (full width at half maximum height of Pv1 band) (Figure S2a-d).

## Discussion

In this study we investigated the effect of osteocyte deficiency induced by overexpression of BCL-2 in osteoblasts and osteocytes on the magnitude of systemic bone loss after fracture. We anticipated that depletion of osteocytes would affect the systemic fracture response due to reduced PLR and altered regulation of osteoblast and osteoclast activity. TG- mice exhibit a similar magnitude of systemic bone loss as previously observed despite differences in strain background (previous studies: C57BL/6; BCL2TG – and + generated from a C57BL/6 x C3H-HEJ hybrid^8^^,^^9^^,^^21^ Overall, our results support our hypothesis that osteocytes deficiency reduced systemic bone loss after fracture, though it remains unclear if osteocytes play a major role in regulating systemic bone loss after fracture. Also osteocyte deficiency was associated with contrary effects in the trabecular and cortical compartments. Consistent with our initial hypothesis, osteocyte deficiency reduced the magnitude of systemic trabecular bone loss in the lumbar spine following femur fracture. Unexpectedly, only TG+ mice exhibited a potential trend toward declines in cortical bone thickness (*p* = 0.087) and significant declines diaphyseal mechanical properties due to fracture. Also fracture did not affect cortical or trabecular lacunar volume. In addition to increased cortical bone volume, osteocyte deficiency also increased whole-bone stiffness, and reduced post-yield displacement.

Our observation of greater bone mass in TG+ mice femora agrees with previous studies demonstrating increased bone mass at 4 mo of age in the appendicular skeleton of BCL-2 TG+ mice.^20^ This has been interpreted as evidence that osteocyte apoptosis enhances osteoblast function and suppresses osteoclast function, since prior work on this mouse model by Moriishi et al. found that bone formation was elevated compared to controls despite similar numbers of osteoblasts, and osteoclastogenesis was inhibited in 4 mo old TG+ mice.^20^ A lack of similarly elevated trabecular bone mass in the lumbar spine suggests that alterations of osteoblast and osteoclast function may not extend to the lumbar spine. Consistent with our prior study, lacunar number per unit volume was also higher in TG+ mice.^32^ While we did not directly evaluate osteocyte death in the current study, our previous TUNEL staining analysis on 12 wk old BCL2 TG+ males of the same age showed that ~60% of osteoctyes were dead.^32^ Thus, despite a greater number of lacuna, the density of viable osteocytes is markedly reduced in TG+ mice.

Expanding upon previous studies of BCL-2 transgene effects on bone, we demonstrate that TG+ bones were stiffer, but also had a lower yield force and considerably decreased post-yield displacement. The increased stiffness and maximum force may reflect enhanced osteoblast function. Moriishi et al. previously demonstrated increased bone formation rate and osteoid thickness in TG+ animals.^20^ Physiological loading induces microdamage.^33^ If not repaired via BMU based remodeling or potentially PLR, damage will accumulate compromising bone strength. Osteocytes orchestrate repair by recruiting bone modeling units, and directly resorbing damaged matrix.^34^ Microdamage can itself induce osteocyte apoptosis, which serves as a recruitment signal for osteoclasts, presumably via the secretion of target signals.^35^^,^^36^ Thus the mass osteocyte apoptosis of BCL-2 TG+ mice may contribute to the lower post-yield displacement or “brittle” phenotype by reducing bone remodeling. As lacunar/canalicular borders represent a large interface for bone removal,^19^ reduced viable osteocyte density and disrupted canaliculi in BCL-2 TG+ mice may prevent the removal of damaged bone via PLR. It is also possible that the greater number of lacunae in TG+ mice in an of itself decreases bone mechanical properties, an interpretation supported by greater lacunar volume fraction in cortical bone in BCL2 TG+ mice. Likewise, our previous study of BCL2 mice indicated the presence of macropores, in BCL2 cortical bone (visible as dark voids in Figure 6B and large vascular canal in S1 this study).^32^ These voids, as well as increased lacunar density, may further contribute to the altered mechanical properties. However, it remains unclear if an increase in macropore size is related to decreases in yield strength in TG+ mice.

The disrupted canalicular network may reduce the production and distribution of cytokines that regulate osteoblast and osteoclast activity including sclerostin, RANKL, OPG, and macrophage colony stimulating factors.^37^ However, we did not detect differences in markers of bone remodeling due to genotype. Given the greater bone mass in TG+ mice, this finding was unexpected. Differences in bone mass may reflect altered remodeling during development that may no longer be occurring in 3-mo-old mice. Also chemical composition of periosteal bone was not altered in TG+ mice, though we did not specifically evaluate this at lacunar/canalicular borders, where the effects of altered PLR may be most visible.^30^

Supporting the hypothesis that disruption of the lacunar/perilacunar network and osteocyte function would reduce the magnitude of systemic bone loss after fracture, TG- mice showed significant declines in lumbar spine trabecular properties, whereas TG+ mice did not. On the other hand, TG+ mice exhibited a trend (0.05 < *p* < 0.1) toward declines in contralateral femoral midshaft cortical thickness and yield force/stress due to fracture, while TG- mice did not. Contrary to our hypothesis that osteocytes PLR plays a major role in systemic bone loss, we detected no differences in lacunar volume due to genotype or fracture. Our prior study using 2D histology also showed that lacunar volume did not differ between fracture and control mice 2 wk post-fracture, whereas canalicular width increased by ~20% in proximal tibia cortical bone.^19^ Combined with the results of the current study, this suggests that, in addition to bone resorption by osteoclasts, fracture upregulates bone resorption by osteocytes at distant skeletal sites, and this occurs primarily along canalicular but not lacunar surfaces at non-injured skeletal sites. The reasons that post-fracture PLR may be confined to canalicular surfaces remains unclear, but the approximately 2.5 greater bone area accessible along canalicular than lacunar surfaces, means more bone surface is available for removal.^38^ Reduced trabecular bone loss in TG+ fracture mice may therefore reflect reduced canalicular remodeling in these regions.

It remains unclear if differences in differences in osteoblast and osteoclast activity also play a role in differences in bone loss between TG+ and TG- animals. TG+ animals have increased osteoblast activity, and osteocyte secreted proteins are key regulators of both osteoblast and osteoclast differentiation and activity. We previously observed that osteoclast number and resorbing bone surface in uninjured femurs was only elevated at 3 d but not 1 or 2 wk post-fracture.^8^ Another group similarly found that osteoclast number in the uninjured femur was not elevated at 2 or 4 wk after femur fracture in mice.^39^ In the current study we evaluated perilacunar and BMU based remodeling indirectly via serum bone remodeling markers. CTX-1 was reduced 5 d post-fracture only in TG- mice, while fracture did not induce any changes in P1NP. While these patterns could explain why less cortical bone loss occurs after fracture in TG- mice, they appear contrary to the trend of reduced trabecular bone loss in TG+ mice. Interestingly, the decrease in bone remodeling markers 5 d post-fracture agrees with our prior data showing reduced expression of key bone metabolism and PLR genes in the contralateral femur of fracture animals including *Mmp2, Sost, Col1a1, Col1a2, Bglap, Bmp1, Alpl, and Mepe* 2 wk after fracture as well as decreased bone formation at 4 to 6 wk after fracture.^19^ This is contrary to prior studies showing a systemic acceleration in systemic bone remodeling after induction of bone defect in rats.^40^ Interpreting these discrepancies is difficult, but changes in serum biomarkers of bone resorption may occur at somewhat different time scales than differences in osteoblast and osteoclast activity or changes in gene expression.

Although BCL-2 TG+ mice showed reduced systemic trabecular bone loss after fracture, we observed a trend (0.05 < *p* < 0.1) toward greater cortical bone loss and reductions in mechanical properties occurred in TG+ mice after fracture. The lack of statistical significance makes interpretations of this pattern tentative. However, trabecular bone remodels at a faster rate due to the greater exposed surface area. Therefore the effect of suppressed osteoclastogenesis and increased osteoblast function in BCL-2 TG+ mice noted by Moriishi et al.^20^ may be more pronounced, resulting in reduced trabecular bone loss compared to TG- mice. The trend toward increased cortical systemic bone loss and decreased mechanical properties in fractured TG+ mice is surprising. It may also relate to compartmental differences in remodeling rate. Potentially signaling molecules can disperse more easily through trabecular bone than cortical bone.^41^

Alternatively, transgene expression may differentially alter signaling pathways in trabecular and cortical bone, suggesting a distinct role for osteocytes in these compartments. There is tentative evidence of this from other studies that also show differential effects of osteocyte signaling on cortical and trabecular bone. For instance, inhibition of osteocyte specific TGF-β signaling increased trabecular but not cortical bone mass in mice. It also decreased cortical bone fracture resistance and mineralization and altered bone chemical composition.^42^ Another study found similar effects following MMP-13 global knockout.^16^ These defects were associated with altered PLR, which differs from the BCL-2 model, in which PLR is largely blocked and osteocyte apoptosis prevents all osteocyte function. Conversely, osteocyte specific deletion of YAP/TAZ reduced trabecular and cortical bone mass as well as bending stiffness.^31^ Similar to the BCL-2 model, YAP/TAZ deletion also increased osteocyte death, but did not alter lacunar volume. Prior review articles have also proposed that osteocyte apoptosis causes an accumulation of mineral in the empty lacunae, leading to a higher stiffness but reduced post-yield displacement, similar to what we observed in BCL-2 TG+ mice.^43^^,^^44^

While osteocyte deficiency alters systemic bone loss after fracture, it did not markedly alter callus size and mineralization. Callus total volume and bone volume did not differ significantly between TG+ and TG- mice. A lack of major differences is somewhat surprising given the documented importance of osteocyte apoptosis and secreted factors such as sclerostin and inflammatory cytokines to well-orchestrated fracture healing.^18^ However, in the current study, we only observed a statistically non-significant trend (*p* = 0.087) toward increased bone mineralization. We have only considered a relatively early (2 wk post-fracture) timepoint. More dramatic differences may be observed at earlier or later time points as the callus becomes more mineralized.

This study has several important strengths. Chiefly, we utilized a unique model of osteocyte deficiency that induces osteocyte death and disrupts the canalicular network.^20^ We also directly visualized the 3D morphology of the lacunar network using XRM and quantified changes in markers of bone turnover as well as bone chemical composition. Lastly, we found novel compartment specific differences in post-fracture systemic bone loss in the context of osteocyte deficiency. However, our study also had several important limitations. We only assessed systemic bone loss and fracture healing at a single time point, and we used only female mice. Also, we assessed microstructure in the femur and osteocyte lacuna in the proximal tibia and we did not directly assess changes in canalicular structure.

In the context of past studies, our findings emphasize the complexity of the role osteocytes play in bone homeostasis, which may differ in trabecular and cortical bone. Some of our findings, primarily reduced trabecular bone loss in BCL2 TG+ mice, support our hypothesis that osteocytes play a significant role in post-fracture systemic bone loss. However, the relatively small difference in other aspects of the systemic response to fracture, eg small differences in microstructural properties and lack of differences in bone remodeling markers between TG+ and TG- mice, also support the null hypothesis that osteocytes do not play a major role in post-fracture systemic bone loss. More studies of both global osteocyte dysfunction and targeted gene manipulation are needed to understand the pathways regulating PLR and osteocyte regulation of bone remodeling both under normal physiological conditions and in response to injury.

## Conclusion

Osteocyte dysfunction induced by overexpression of human BCL-2 in osteoblasts and osteocytes increased bone volume in the appendicular skeleton. While stiffness was increased, post-yield displacement decreased. Also BCL-2 TG+ mice exhibited reduced systemic trabecular bone loss after fracture compared to TG- littermates, and there was a trend (0.05 < *p* < 0.1) toward increased cortical bone loss in TG+ fracture mice. Given the extent of osteocyte depletion in the BCL-2TG+ mice, greater differences would have been expected if osteocytes were the primary drivers of systemic bone loss. Thus, these results may also support the null hypothesis that osteocytes do not play a significant role in mediating systemic bone loss after fracture. More studies are needed to ascertain the role of osteocytes in the skeletal response to fracture, either through direct resorption of bone matrix, especially at the canalicular surfaces, or through modulation of osteoblast and osteoclast activity.

## Supplementary Material

Supplementary_Figure_1_ziae135

S2_ziae135

## Data Availability

All data presented in the manuscript are available per reasonable request.
