# Supplementary figures and images for: Altered post-fracture systemic bone loss in a mouse model of osteocyte dysfunction
Source: JBMR Plus. 2024 Nov 1;8(12):ziae135. doi: 10.1093/jbmrpl/ziae135 (PMC11601886; doi:10.1093/jbmrpl/ziae135)

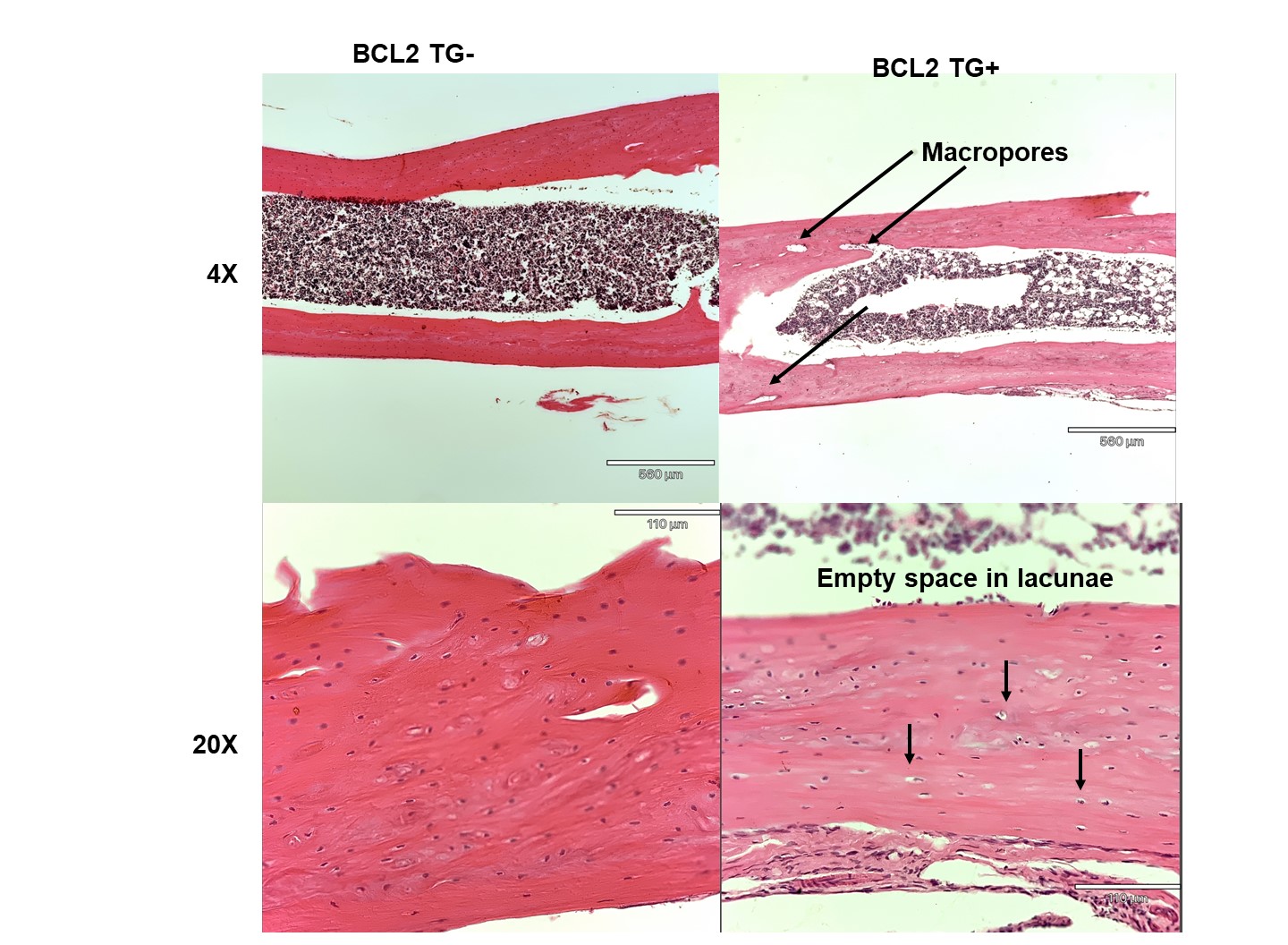

Supplement: Supplementary_Figure_1_ziae135 [file supplementary_figure_1_ziae135.jpeg]

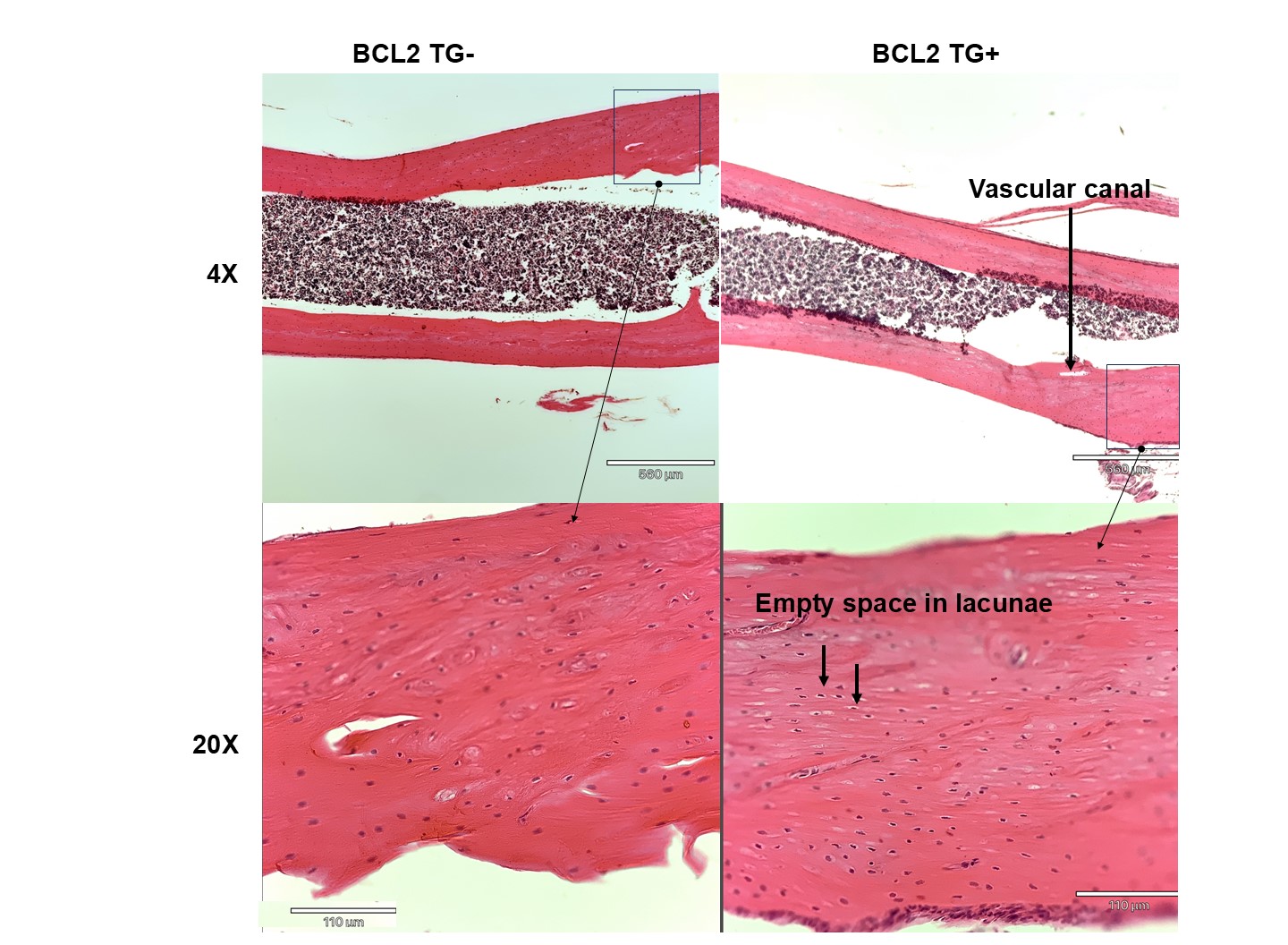

Supplement: S2_ziae135 [file s2_ziae135.jpeg]
